# Tryptophan: From Diet to Cardiovascular Diseases

**DOI:** 10.3390/ijms22189904

**Published:** 2021-09-14

**Authors:** Nada Joe Melhem, Soraya Taleb

**Affiliations:** Paris Cardiovascular Research Center, Institut National de la Santé et de la Recherche Médicale (Inserm), Université Paris-Descartes, 75015 Paris, France; nada-joe.melhem@inserm.fr

**Keywords:** tryptophan, tryptophan catabolism, kynurenine, IDO, cardiovascular disease, atherosclerosis, myocardial infarction, cardiometabolic diseases

## Abstract

Cardiovascular disease (CVD) is one of the major causes of mortality worldwide. Inflammation is the underlying common mechanism involved in CVD. It has been recently related to amino acid metabolism, which acts as a critical regulator of innate and adaptive immune responses. Among different metabolites that have emerged as important regulators of immune and inflammatory responses, tryptophan (Trp) metabolites have been shown to play a pivotal role in CVD. Here, we provide an overview of the fundamental aspects of Trp metabolism and the interplay between the dysregulation of the main actors involved in Trp metabolism such as indoleamine 2, 3-dioxygenase 1 (IDO) and CVD, including atherosclerosis and myocardial infarction. IDO has a prominent and complex role. Its activity, impacting on several biological pathways, complicates our understanding of its function, particularly in CVD, where it is still under debate. The discrepancy of the observed IDO effects could be potentially explained by its specific cell and tissue contribution, encouraging further investigations regarding the role of this enzyme. Thus, improving our understanding of the function of Trp as well as its derived metabolites will help to move one step closer towards tailored therapies aiming to treat CVD.

## 1. Introduction

Cardiovascular disease (CVD), including myocardial infarction (MI) and stroke, constitutes the leading cause of death worldwide. Atherosclerosis is the underlying cause of the majority of CVD. Despite the use of cholesterol-lowering therapies to reduce atherosclerosis, nearly 18 million people die each year due to CVD [1]. Given the high prevalence of obesity in developing countries, the global incidence of CVD is predicted to increase and constitute the greatest public health problem in the world.

Atherosclerosis is a chronic inflammatory disease affecting both large and medium arteries initiated by increased endothelial cell (EC) permeability and intimal low-density lipoprotein (LDL) cholesterol accumulation [2]. Over years, atherosclerotic plaque overgrowth can lead to plaque instability and a high probability of rupture. The disruption of the plaque surface and exposure of its thrombogenic content to the luminal blood flow result in thromboembolism, which may occur in one or more coronary arteries, contributing to their occlusion [2]. Therefore, blood oxygen and nutrient deprivation result in ischemia, and consequently myocardial cell death, contributing to acute MI most commonly occurring in the left ventricle (LV) [3,4]. MI is therefore considered as the first and most severe clinical manifestation of atherosclerosis.

Inflammation was identified as a key driver of atherothrombotic events [5,6,7]. On this subject, the authors of the Canakinumab Anti-Inflammatory Thrombosis Outcomes Study (CANTOS) trial showed that targeting interleukin-1β (IL-1β) improved CV outcome in patients with MI history, which would confirm the proof of concept of the role of inflammation in the causal pathway of atherothrombosis disease [8]. This was followed by the Low-Dose Colchicine after Myocardial Infarction (COLCOT) trial which revealed that non-selective inhibition of inflammation using colchicine significantly reduced atherosclerosis-related events [9].

Several actors can contribute to inflammatory-associated response which include modifiable and non-modifiable risk factors. Among modifiable risk factors, diet and particularly its composition has been suggested to significantly impact inflammation. In this regard, some amino acids (AAs) metabolic pathways have become critical checkpoints for controlling inflammatory-related mechanisms. For instance, branched-chain AAs (leucine, isoleucine, and valine) have been shown to promote EC dysfunction through increased reactive oxygen species (ROS) generation and inflammation [10]. Likewise, tryptophan (Trp) metabolites have been shown to be closely related to inflammation and thereby suggested to be involved in CVD [11]. In that respect, some systemic Trp catabolites were associated with worse CVD outcome and have been considered as important predictors of major acute coronary events [12,13,14,15], as well as biomarkers of adverse LV remodeling following MI [16].

In this review, we discuss our growing appreciation of Trp absorption and metabolism through the action of different enzymes, mainly through indoleamine 2, 3-dioxygenase 1 (IDO), as well as their involvement in CVD. We discuss known and potential mechanisms of action of Trp catabolites in an array of CV-related diseases and their relevance as putative therapeutic targets.

## 2. Trp Absorption and Catabolism

Trp was discovered by the English chemist Sir Frederick Gowland Hopkins in 1901 [17]. It is one of the nine essential amino acids that cannot be synthesized endogenously and is therefore supplied by the diet. Daily consumption of Trp is about 900–1000 mg, a level which is high considering the WHO recommendation of about 4 mg/kg (~300 mg for an adult). Trp is found in protein-rich foods, such as meat, fish, eggs, milk, cheese, dairy foods, beans, nuts, pumpkin, sesame seeds, turkey, tofu, soy, and chocolate [18].

Following food digestion, Trp absorption occurs through B^0^AT1 and TAT1, located respectively in the apical and basolateral membranes [19,20]. Some intestinal Trp enters portal and then systemic circulation after absorption and the remaining Trp is locally transformed, notably by gut microbiota into various catabolites.

**Figure 1:**
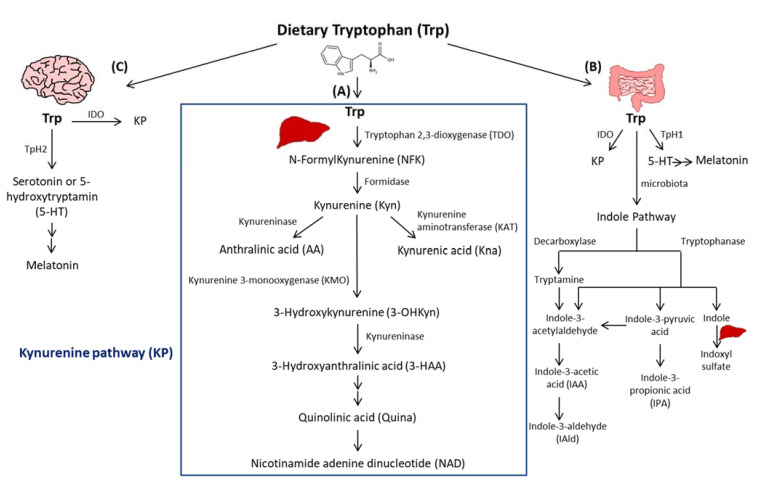
Simplified illustration of the tryptophan degradation in different peripheral organs. Tryptophan (Trp) is predominantly converted into kynurenine (Kyn) pathway (KP) by Tryptophan 2, 3-dioxygenase (TDO) in the liver (**A**) and by indoleamine 2, 3-dioxygenase 1 (IDO) in extrahepatic organs. In the gastrointestinal tract, a small amount of Trp is converted by gut microbiota through the action of the enzymes tryptophanase and decaboxylase, into indole and its derivatives and into tryptamine (**B**). Indole metabolites could be converted in the liver into indoxyl sulfate. Another fraction of Trp is converted through Trp hydroxylase 1 (TpH 1) into serotonin in the gut (**B**) and through Tph2 in the brain (**C**) then to melatonin.

Trp passes into the hepatic portal system before the unused fraction is released into the bloodstream and delivered to peripheral tissues. In addition to protein synthesis, which represents a small portion (1%), Trp mostly undergoes degradation, a process known as Trp catabolism (Figure 1). Trp-derived metabolites are generated through three known pathways orchestrated by tissue-specific enzymes. The Kynurenine pathway (KP) accounts for ~95% of ingested Trp [21]. Under normal physiological conditions, the first and rate limiting step of Trp catabolism is mostly catalyzed by tryptophan 2, 3-dioxygenase (TDO), mainly expressed in the liver. Its activity is constitutive but could be increased by glucocorticoids and circulating levels of Trp [22]. A minimal contribution is also possible due to the extrahepatic cytosolic enzyme, IDO, and to a lesser extent by its isoform, the indoleamine 2, 3-dioxygenase 2 (IDO2). As a result of TDO, IDO, or IDO2 activity, N-formylKynurenine (NFK) is generated and hydrolyzed to Kyn by NFK formidase. Kyn is then catabolized to several metabolites via the action of multiple enzymes. Kyn is converted to anthranilic acid (AA) by kynureninase, or kynurenic acid (Kna) by kynurenine aminotransferase (KAT), and to 3-hydroxykynurenine (3-OHKyn) by kynurenine monooxygenase (KMO). Then, 3-OHKyn is metabolized into 3-hydroxyanthranilic acid (3-HAA) by kynureninase. As final steps, quinolinic acid (Quina) is generated, and thereafter nicotinamide adenine dinucleotide (NAD), which plays an essential role in energy metabolism. The extrahepatic KP is minimal (<2%) under homeostasis, but becomes quantitatively more significant under inflammatory conditions, when the activity of IDO exceeds that of hepatic TDO [22].

The second pathway of Trp degradation is the serotonin pathway (~1–2%) mediated by Trp hydroxylase (TPH) that results in the generation of serotonin or 5-hydroxytryptamin (5-HT), the precursor of melatonin [21,23]. The two enzymes involved in Trp hydroxylation are TPH1, mainly expressed in the enterochromaffin cells in the gut [24], and the TPH2 preferentially expressed in the brain [25]. TPH1, whose activity is in part modulated by gut microbiota [26], is responsible for the generation of more than 90% of 5-HT of the body [26]. The serotonin from the gut can enter the circulation or the intestinal lumen to exert its action on almost all the organs. It regulates gut motility, vascular tone, primary hemostasis, and cell-mediated immune response [27]. In the central nervous system, 5-HT generated due to TPH2, controls mood, sleep, anxiety, and food intake. Interestingly, since deregulated TPH2 activity has been implicated in psychiatric disorders, it has been considered as a therapeutic target for these diseases [28]. Of note, serotonin reuptake inhibitors (SSRIs) are considered the most used anti-depressant drugs. Besides their role in blocking 5-HT reuptake transporters in the brain, SSRIs are responsible for 5-HT storage depletion in the platelets, which are the major storage cells for peripheral 5-HT. Melatonin results from 5-HT catabolism in mainly pineal gland in which the peak of its production and release happens during night. It is known to regulate sleep and circadian rhythms, and to act as a free radical scavenger as well as a suppressor of inflammation [23].

In the gastrointestinal tract, Trp is mostly converted in the host to Kyn by IDO, and to a lesser extent to 5-HT, and to indole metabolites by the microbiota [21]. Trp catabolism has a major role in fine-tuning intestinal physiology that may affect peripheral health [29]. In this context, it has been shown that aberrant Trp metabolism and gut microbiota interplay contributes to immune response activation in experimental systemic lupus erythematosus [30]. Consistently, mice lacking dietary Trp displayed an impaired intestinal immunity and microbial dysbiosis [31]. On the other side, germ-free mice, which lack an intestinal microbiome, exhibited reduced Kyn levels, highlighting the importance of gut microbiota for the induction of IDO activity [32].

Among Trp catabolites, indoles play a pivotal role in human physiology, contributing to several functions, such as metabolic homeostasis, and immune system maturation and stimulation [33]. Some bacteria express enzymes responsible for Trp catabolism, more specifically decarboxylase and tryptophanase. Their action leads to Trp conversion into tryptamine and indole metabolites, including indole pyruvic acid, indole propionic acid (IPA), indole acetaldehyde, indole acetic acid (IAA), indole aldehyde (IAld), and indoxyl sulfate in the liver [21]. Indoles are key effectors in the gut, and they are known to be endogenous ligands of the Aryl Hydrocarbon Receptor (AHR) [21]. AHR is a transcription factor principally known to control environmental toxicity by binding exogenous xenobiotic toxic chemicals [34]. It also conveys microbiota-generated indole protective effects through antimicrobial peptide (AMP) production and mucosal protection from inflammation through IL-22 production [35,36]. Accordingly, IPA, an indole metabolite has been shown to improve intestinal barrier function through activation of the Pregnane X Receptor (PXR) [37]. Trp catabolism is therefore a key modulator of host–gut microbiota crosstalk and any perturbation impacting this mutualistic relationship can lead to disease development. For instance, a deficiency in the host gene caspase recruitment domain family member 9 (*Card9*), involved in the immune response against microorganisms, was shown to alter the microbiota composition and function, failing to produce indoles and contributing to intestinal inflammation [36]. Moreover, the disequilibrium between Kyn and indole production has been shown to affect metabolic and intestinal diseases, such as obesity [38] and celiac disease [39]. Increasing evidence has suggested a strong relationship between gut microbiota and its derived metabolites with CVD [40]. In this regard, patients with inflammatory bowel disease (IBD) have an increased risk of atherothrombotic complications [41], suggesting a link between the intestine and the cardiovascular system. Given the importance of Trp metabolites in gastrointestinal homeostasis, they may play a significant role in CVD. In this regard, patients with chronic kidney disease exhibit an accumulation of indoxyl sulfate due to insufficient renal removal, suggested to be involved in the occurrence of CVD in these patients through enhanced oxidative stress [42]. Moreover, indoxyl sulfate has been shown to induce vascular smooth muscle cell (SMC) proliferation that may sustain neointimal formation during uremia [43]. However, the causative relationship between indoxyl sulfate and CVD has not yet been fully proven and the plasma levels of indoxyl sulfate achieved in chronic kidney disease are supraphysiological, which may not be representative of what occurs in CVD patients without severe renal insufficiency. On the other hand, certain indole metabolites seem to exert anti-inflammatory effects [44]. Therefore, future studies are needed to investigate the contribution of intestinal Trp metabolites in CVD.

## 3. Indoleamine 2, 3-Dioxygenase 1

IDO is a rate limiting enzyme implicated in Trp catabolism via KP. It was discovered in rabbit small intestine by Hayaishi and colleagues in 1967 [45,46]. IDO is a monomeric protein of ~45 kDa constituted of 403 AAs and encoded by *Ido-1* gene located in a pericentromeric region on chromosome 8.p12-p11 in humans and 8 A2 in mice. It is a widely expressed non-secreted cytosolic enzyme [47]. When produced, it exists in an inactive, heme-free, apoenzyme form. It becomes an active holoenzyme after the binding of heme, superoxide anion, and its substrate L-Trp [48].

Trp catabolism was originally proposed to be an innate immune defense mechanism to protect the host against bacterial or viral infection. Initial studies conducted by Pfefferkon et al. showed that interferon-γ (IFN-γ)-induced Trp degradation by human fibroblasts was responsible for blocking the intracellular parasite *Toxoplasma gondii* growth in these cells [49]. It was also considered as a central mediator of immune tolerance in pregnancy and a regulator of adaptive immune response [50]. On the same note, macrophage Trp catabolism was shown to inhibit T cell proliferation [51]. Later, it was shown that these immunoregulatory effects rely on the depletion of Trp in the microenvironment and/or the generation of biologically active metabolites.

Data from literature show a complex role of tissue-specific IDO activity involved in the generation of Trp metabolites, which could have either deleterious or protective effects in different pathological settings. Moreover, the discrepancy of the observed roles of IDO could be potentially explained by tissue-specific effects of this enzyme, suggesting that its function is tailored to the tissue and the environmental cues.

In recent years, IDO has been implicated in the pathophysiology of several inflammatory diseases, including infection, allergy, autoimmunity, chronic inflammation, inflammatory neurologic diseases, transplantation, and cancer [52]. The involvement of IDO in such diverse diseases is due to its large spectrum of expression in different organs and tissues, such as the spleen, brain, intestine, and lung [47].

During inflammation, IDO is up-regulated mostly in macrophages and dendritic cells by pro-inflammatory stimuli, such as IFN-γ [53]. IDO is an upstream enzyme which is involved in the generation of Kyn, and thereafter, due to different downstream other enzymes, the generation of Kyn-derived metabolites that have been described to exert either protective or deleterious effects [54,55]. For example, Quina plays a direct role in the pathogenesis of neurodegenerative disorders through the activation of *N*-methyl-d-aspartate (NMDA) receptors [54,56]. In contrast, Kna is neuroprotective through its antagonism on NMDA receptors [57].

As a protective role, IDO has been described as immunosuppressive enzyme that suppresses effector T Helper (TH17) -cell function and favors the differentiation of regulatory T cells (Tregs) [52]. Some KP-derived metabolites, particularly Kyn and Kna, were identified as endogenous ligands of AHR [58,59]. As such, Kyn was shown to cross the T-cell membrane by the system L-type amino acid transporter SLC7A5 before it activates the AHR by binding to its hydrophobic ligand-binding pocket [60]. Recently, two trace-active derivatives of Kyn named trace-extended aromatic condensation products (TEACOPs) have been identified and considered as high affinity AHR ligands [61]. Moreover, IDO expression was shown to be maintained due to an IDO-Kyn/AHR-IDO feed-forward loop [62]. IDO-mediated effects also rely on Trp consumption. Notably, Trp depletion induces the stress-response enzyme, general control nondepressible 2 (GCN2) kinase, which inhibits the anabolic mammalian target of rapamycin (mTOR) [63]. GCN2 activation in T cells can inhibit their proliferation and promote T cell differentiation toward Treg cells. GCN2 can also directly affect the phenotype of dendritic cells and macrophages by inducing an anti-inflammatory response [64].

Independently of its enzymatic activity, IDO can act as a signal transducer conferring a tolerogenic phenotype to plasmacytoid dendritic cells (pDCs) in transforming growth factor (TGF)-β-dependent manner [65]. This regulatory role of IDO may be highly relevant in the context of acquired peripheral tolerance, e.g., in pregnancy, graft tolerance, cancer, chronic infection, autoimmunity, and allergic inflammation [66].

In addition, the biological effects of IDO may go beyond its role in the regulation of the immune response, suggesting a more complex and prominent role than previously thought. IDO activity was shown to contribute to arterial vessel relaxation and to the control of blood pressure in septic shock [67]. Moreover, metabolites generated from Kyn may regulate diverse cellular functions, including viability [68], adhesive, and migratory properties [69], as well as inflammatory potential. As such, Trp metabolism has been involved in various diseases, ranging from chronic granulomatous disease [70] to neurodegenerative diseases, such as Alzheimer’s or Huntington’s disease [54]. Despite the progress made to understand the modes of action of IDO, more studies are warranted to comprehend the profile of IDO expression and activation as well as its precise role in immune-dependent diseases.

## 4. Trp Catabolism in Cardiometabolic Diseases

**Figure 2:**
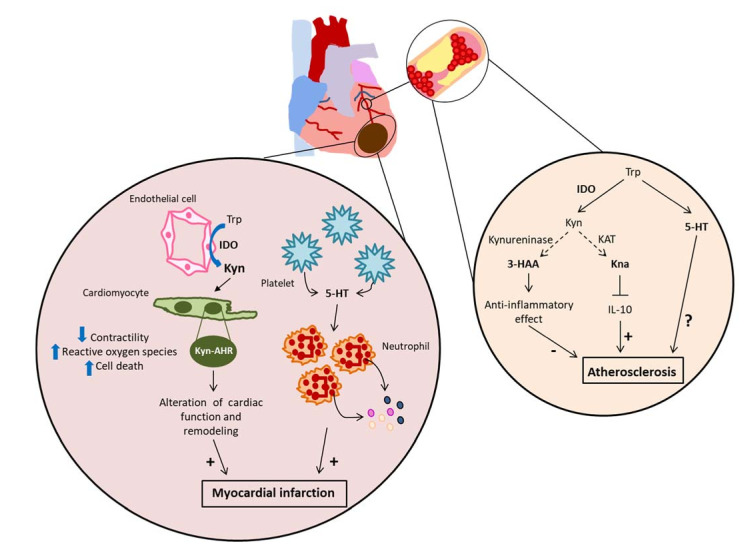
Potential mechanism of actions of Trp metabolites in CVD. In ischemia-induced myocardial infarction (MI), induction of IDO in endothelial cells is responsible of paracrine effects on cardiomyocyte apoptosis through an aryl hydrocarbon receptor (AHR)-dependent manner, as well as an alteration of cardiomyocyte contractility. This impacts on cardiac remodeling and function. In MI, serotonin (5-HT) produced by platelets activates neutrophil degranulation, which aggravates MI. In atherosclerosis, Kyn-derived metabolites, 3-HAA (hydroxyanthranilic acid) has been shown to exert protective effect, whereas another Trp-derived metabolite, kynurenic acid (Kna) has been shown to have deleterious effects on plaque development through the down-regulation of the anti-inflammatory cytokine, IL-10.

The strong entanglement between IDO and inflammation, along with clinical data that suggest a link between IDO and cardiovascular diseases, prompted research on the role of this enzyme in CVD, particularly atherosclerosis. Besides, emerging data show its implication in CVD-associated comorbidities such as obesity. The bulk of these data suggest a rather pathogenic role of IDO in CV-related diseases, such as stroke [71,72], aneurysm [73,74], and MI [75], whereas its role in atherosclerosis is still under debate [76,77,78,79] (Figure 2).

### 4.1. Metabolic Syndrome

In obesity, IDO activity as assessed by Kyn/Trp ratio is increased in adipose tissue, circulating blood, and likely in the digestive tract of obese compared to non-obese subjects [38,80,81,82,83]. An emerging concept suggests that Trp-catabolism via IDO expressed in the gastrointestinal tract could significantly contribute to metabolic disease. Our study based on the use of obese mouse model demonstrated the implication of the enzymatic activity of IDO in metabolic syndrome [38]. We have shown that the deletion or inhibition of IDO improved insulin sensitivity, decreased endotoxemia and chronic inflammation, and positively regulated lipid metabolism in the liver and adipose tissues. We found that these beneficial effects were due to Trp metabolism deviation from Kyn pathway toward Trp metabolism by the gut microbiota and the production of IL-22 effects that were abrogated after administration of a neutralizing anti-IL-22 antibody. In addition, microbiota transfer from obese mice treated with IDO inhibitor compared to untreated mice increased the production of IAA as well as IL-22 and improved metabolic parameters in recipient mice fed a high fat diet [38]. Moreover, we and others have shown that indole metabolites, in particular IAA, protected against complications of metabolic syndrome [38,84], illustrating the importance of Trp metabolites for intestinal homeostasis and peripheral metabolism. The protective role of indoles can be linked to their local effects on the intestine by promoting the production of IL-22 and/or the stimulation of enteroendocrine L cells, inducing the production of GLP-1 (glucagon-like peptide-1), an incretin stimulating insulin secretion by pancreatic β cells [84,85]. In addition, indole has been shown to reduce liver inflammation in mice by preventing the harmful effects induced by lipopolysaccharide (LPS) [86]. In this regard, supplementation with bacteria of the Lactobacillus strain producing indoles led to the improvement of metabolic parameters, maintaining intestinal barrier function and promoting GLP-1 production [84]. Therefore, these results suggest a beneficial local effect of Trp-derived indoles on intestinal homeostasis, also impacting the periphery, in particular by improving metabolic parameters.

On the other hand, administration of Trp-derived metabolite Kna to wild-type (WT) mice has been shown to activate the G protein-coupled receptor (GPR) 35, known to be a Kna receptor [87], and to increase energy expenditure [88], which suggested a protective effect of KP. However, this may not represent the role of endogenous Kna with physiological concentrations, as this experiment was performed in wild-type (WT) mice.

Besides, mouse models of obesity have shown that serotonin produced by the intestine promoted metabolic syndrome by downregulating thermogenesis of brown adipose tissue [89]. However, this may be questionable in humans since the notion of brown adipose tissue dependent energy expenditure change in adults is still under debate. Human data revealed a dysregulation of Trp metabolites with an increase in host-dependent Kyn levels in feces along with a decrease in gut microbiota-dependent indoles [38,84].

Taken together, these data strongly suggest a protective role of gut microbiota-produced indoles in the gastrointestinal tract which beneficially affect peripheral metabolism. However, the role of the other Trp-derived metabolites, particularly Kyn and its derived metabolites in metabolic diseases is still under-investigated.

### 4.2. Abdominal Aortic Aneurysm

Abdominal aortic aneurysm (AAA) is one of the leading causes of cardiovascular death being fatal in 80% of the cases when ruptured. This disease mainly affects adults aged more than 65 years [90]. It is principally mediated by Angiotensin II (AngII) and defined as a permanent localized dilation of the abdominal aorta. The mechanisms involved in AAA formation are elastin degradation, SMC apoptosis, compensatory collagen deposition, oxidative stress, and chronic aortic wall inflammation [91,92].

The control of blood pressure has a major impact on the clinical course of AAA. A previous study attributed Trp catabolism by IDO to the control of vascular tone. Particularly, the induction of IDO in ECs, resulting in Kyn generation, contributes to arterial vessel relaxation in an LPS-induced endotoxin shock mouse model. The Kyn attributed effect was due to adenylate and guanylate cyclase activation [67]. On the other hand, nitric oxide (NO), which also mediates vessel relaxation, has been shown to inhibit IDO [93]. Indeed, IDO-mediated Kyn production may be a “back-up” system contributing to vessel relaxation in situations where activation by NO is absent. This effect may have a major contribution to the microvascular reactivity in conditions, like myocardial infarction, sepsis, and stroke. Nevertheless, the potential vasodilator role of IDO is not consistent with experimental and human data showing an activation of KP post-stroke, which was associated with altered cognitive function and mortality [94]. Besides, some experimental data showed a rather deleterious role of the KP in AAA.

In one study, silencing kynureninase, which was upregulated in macrophages in the aneurysmal walls in patient suffering from AAA, increased the expression of IL-6 and IDO in cultured macrophages, suggesting an anti-inflammatory effect of macrophage kynureninase [95]. In contrast to this study, another finding highlighted a role of the KP catabolite, 3-OHKyn, on EC apoptosis and dysfunction through the generation of superoxide anions by nicotinamide adenine dinucleotide phosphate (NAD(P)H) oxidase [96]. The direct role of KP in AAA has been demonstrated through the implication of IDO and the Trp-derived metabolite, 3-HAA [73]. Mechanistically, AngII-induced AAA was associated with an increased IFNγ level resulting in an elevated IDO and the downstream kynureninase expression, which is directly responsible for 3-HAA generation. In turn, 3-HAA up-regulates matrix metallopeptidase 2 via the activation of nuclear factor-κB (NF κB). Consistently, human aneurysmal aortic sections exhibit stronger 3-HAA, IDO, and kynureninase staining than in adjacent nonaneurysmal aortic sections [73]. On the same note, our group has shown that *Ido-1* knock-out (KO) protected Low-density lipoprotein receptor deficient mice (*Ldlr^−/−^*) fed a high cholesterol (HC) diet against AngII-induced AAA through the prevention of vascular SMC apoptosis [74]. These findings have been instructive in delineating basic principles that might underlie AAA development in humans. However, more mechanistic studies are expected to further dissect the underlying Trp metabolic effects in AAA.

### 4.3. Atherosclerosis

Atherosclerosis is a lipid-driven chronic inflammatory disease mediated by many immune cells involved in different steps of disease, including initiation and progression [97,98]. In the early stages of the disease, both innate (i.e., DCs, macrophages, and neutrophils) and adaptive immune cells (B and T lymphocytes) contribute to the modulation of the inflammatory response. Early investigations regarding a potential role of IDO in atherosclerosis were rather indirect. Daissormont et al. reported a protective effect of pDCs in a model of atherosclerosis and proposed that it might be related to IDO activity [99]. Moreover, a more recent study suggested that IDO-expressing aortic pDCs protect against atherosclerosis through induction of Treg cells [100]. However, both studies provide no demonstration of a direct effect of IDO expression by pDCs on Tregs in vitro or in vivo. In this later study, they demonstrated a reduced Tregs number in IDO-deficient mice, which is not pDC restricted, and therefore the potential contribution from other cell types expressing IDO cannot be ruled out. Moreover, unlike the previous study showing a protective role of pDC in atherosclerosis [99], a more recent finding suggested a pro-atherogenic role of pDCs [101].

Concerning the inhibition of IDO activity, in one study, administration of IDO inhibitor 1-methyl-tryptophan (1-MT) to *Ldlr^−/−^* mice did not significantly alter lesion formation [76]. Moreover, 1-MT administration did not alter Kyn levels. It was later shown that the administration of the KP-catabolite, 3-HAA, reduced total plasma cholesterol and triglycerides levels as well as atherosclerotic lesion area in the aorta of HC diet-fed *Ldlr^−/−^* mice. This was associated with adaptive immune response modulation and systemic inflammation reduction [102]. Based on these findings, 3-HAA was considered as a novel anti-atherosclerotic agent having beneficial effects on lipid metabolism and vascular inflammation. However, this study remained inconclusive since the observed effects were obtained with supra-physiological levels of 3-HAA, which does not reveal its endogenous role. In addition, the observed protective effects were most likely due to the profound reduction of plasma cholesterol and triglycerides levels rather than to a direct immunomodulatory role of 3-HAA. Given that kynureninase directly controls 3-HAA production, it would be interesting to inhibit or invalidate this enzyme to examine the role of endogenous 3-HAA in atherosclerosis.

Another study showed that by inhibiting IDO with 1-methyl-DL-tryptophan (D/L 1-MT), apolipoprotein e (*Apoe)^−/−^* mice developed bigger atherosclerotic lesions in the aortic arch and root as well as an enhanced vascular inflammation associated with an up-regulation of the vascular cell adhesion molecule-1 (VCAM-1), macrophage chemotactic protein CCL2, and an increased CD68-macrophage infiltration into the intima of the vessel wall [77]. However, no significant difference was observed in plasma Kyn/Trp ratio after D/L 1-MT administration, indicating inefficient IDO activity inhibition and suggesting that the pro-atherogenic effect could be due to the significant increase in plasma total cholesterol and the significant decrease in high density lipoprotein (HDL)-cholesterol observed in the group treated with D/L 1-MT. Another group showed that IDO deficiency in *Apoe^−/-^* mice with small plaques had a pro-atherogenic role [78]. The effect was restricted to the development of small fatty streaks and did not impact advanced atherosclerotic lesions. In contrast, our group found that a marked decreased Kyn/Trp ratio, through the use of IDO-deficient *Ldlr^−/−^* mice, led to an increase in IL-10 [79], a major immune-regulatory and athero-protective cytokine, and to atheroprotection. We showed that this mechanism was important to maintain immune tolerance observed in IDO-deficient mice. Indeed, double deficiency IDO/IL-10 led to exaggerated immune responses and these mice developed severe spontaneous colitis and accelerated atherosclerosis. We demonstrated that IDO activity was required for the regulation of IL-10 through the generation of downstream Kna. Consistently, supplementation of IDO-deficient mice with physiological levels of Kna limited IL-10 expression and promoted atherosclerosis. The finding that IDO activity leads to IL-10 inhibition is intriguing and is supported by other observations. For example, pharmacologic inhibition or genetic deletion of *Ido-1* reduced the inflammatory response to LPS in vivo as revealed by reduced TNF-α, IL-6, and IL-12 and increased IL-10 [103]. Furthermore, activated B cells from *Ido-1^−/−^* mice were shown to produce more IL-10 [104], which is in contradiction with Cole et al. results [78]. Moreover, Von Bubnoff and colleagues showed that human IDO-positive DC populations produced significantly more IL-1β and IL-15 and less IL-10 and IL-6 than the IDO-negative subset after CD40L stimulation [105], which supports the role of IDO in restraining IL-10 production. This seems to be specific for IDO, as double deficiency of IDO and IDO2 did not show the same result [106]. Consistent with our results regarding the negative regulation of Kna on IL-10, a more recent paper showed that the induction of IDO by oxazoles reduced IL-10 production in intestinal cells [107]. This mechanism was dependent on the production of Kyn metabolites such as Kna, which inhibited IL-10 production through AHR mediated-intestinal inflammatory response. Interestingly, human data revealed that plasma Kyn/Trp ratio was found to be positively associated with LDL cholesterol and triglycerides, as well as carotid intima-media thickness in individuals with advanced atherosclerosis [12]. Moreover, an association has been deduced between increased levels of Kna and an unstable plaque phenotype in patients with coronary artery diseases [79]. In contrast to these results, a more recent study showed an upregulation of enzymes within Quina branch, along with a decrease in Kna pathway in human carotid plaques in comparison to control aorta [108]. However, this concerns enzyme expression and not activity. More recently, the same group showed that the deletion of Kna receptor GPR35 did not play a direct role in macrophage activation, vascular inflammation, and the development of atherosclerosis [109].

The reason for the discrepancy of IDO function is unclear and may be related to the used mouse model *Ldlr^−/−^* vs *Apoe^−/−^*, the diet, and the nature of Trp-derived metabolite involved in the phenotype. Thus, further studies using a cell-type specific IDO KO as well as invalidation of the enzymes downstream of IDO are needed to clarify the role of KP in atherosclerosis.

In addition to the important role exerted by the KP in atherosclerosis, evidence suggests that Trp catabolism through the 5-HT pathway could be also considered as an important player in this pathology. Interestingly, treatment of *Apoe^−/−^* mice with SSRI increased atherosclerotic lesion formation associated with enhanced vascular permeability and elevated chemokine-induced integrin-binding activity of circulating leukocytes as well as increased myeloid cell adhesion in the carotid arteries [110]. In this study, the proatherogenic effect of SSRI seems to occur independently of peripheral 5-HT depletion given that no atherosclerosis aggravation has been shown following TPH1 inhibition at a late stage. More mechanistic studies are expected to examine the 5-HT involvement in atherosclerosis.

Human studies highlighted a role of 5-HT in predicting CAD and the occurrence of cardiac events in patients undergoing coronary angiography for chest pain or clinically suspected angina pectoris [111]. In another cohort, authors showed that circulating 5-HT levels were significantly correlated with derivative reactive oxygen metabolites and the monoamine levels were more important in CAD patients compared to non-CAD subjects [112].

Melatonin which is a metabolite produced downstream of 5-HT has also been involved in atherosclerosis. Melatonin exerts its biological role through binding to membrane and nuclear receptors. Membrane receptors include the melatonin receptor and among nuclear receptors are members of the retinoic acid related orphan receptor/retinoic acid Z receptor (ROR/RZR) family [113].

Hu and coworkers showed that the melatonin treatment of high-fat-fed rabbits improved vascular endothelial dysfunction and inflammation as well as reduced atherosclerosis [114]. Another more recent study showed that melatonin administration to *Apoe^−/−^* mice with a rupture-prone vulnerable carotid plaque induced by endogenous renovascular hypertension combined with low shear stress prevented plaque rupture and decreased inflammation in intraplaque macrophages in a RORα-dependent manner [115].

Further investigation should be undertaken in order to understand the impact of not only Kyn, but also Kyn-derived metabolites as well the other Trp-dependent metabolites in atherosclerosis. With this objective, targeting the Trp catabolism pathway could constitute a beneficial, holistic anti-inflammatory strategy for patients suffering from atherosclerosis.

### 4.4. Acute Myocardial Infarction

Acute MI is mainly the complication of atherosclerotic plaque rupture or erosion, and coronary artery occlusion by a thrombus. During acute MI, loss of myocardial cells is associated with a process of healing to preserve LV shape and function [116]. This reparative response consists of inflammation and fibrosis playing an essential role during this phase, aiming to clean the damaged site from dead cells and form a firm scar. However, in some pathological conditions, an impaired healing could be deleterious and results in adverse LV remodeling. This latter involves changes in the form and function of the LV including dilatation, infarct expansion, and increased likelihood of heart failure (HF) [117].

Over the years, the perception of how patients with MI should be treated has been substantially improved thanks to surgical revascularization and percutaneous coronary intervention thus, contributing to a significant decrease in mortality [118]. Even with all these advances, many uncertainties exist given that there is, to date, no biomarker able to identify the patients at risk of adverse LV remodeling responsible for the high HF prevalence [119]. Moreover, many therapies failed to show cardioprotection and no treatment could repair cardiac damage and reduce infarct size [4]. Cumulative evidence from human and experimental studies indicates that inflammation plays a crucial role in post-ischemic cardiac tissue remodelling. Then, factors which impact on inflammation may represent potential targets to reduce MI.

Previous cohorts have relied on Trp catabolites and plasma or urine Kyn/Trp ratio as markers positively associated with major coronary events, MI, and mortality in patients with or without a known history of coronary heart disease (CHD) [12,13,14,15,16,79]. However, one observation ascertained that IDO was inversely linked to CHD in healthy participants of European descent [120]. Surprisingly, IDO was analyzed in the plasma, which is implausible given that this enzyme is known to be intracytoplasmic. Importantly, these human studies strongly suggest that the role of IDO in MI is likely to be prominent, but this needs to be experimentally tested.

In fact, experimental studies aiming for a deeper understanding of cellular and molecular mechanisms underlying MI are still lacking. Notably, communication between the different cardiac cell populations is crucial to perform the essential function of the heart and loss of cells during MI deregulates cell interactions and leads to harmful consequences [121]. This also raises the question of whether MI-associated complications result from activation of specific cells primed to respond to injury. In the heart, cardiomyocytes (CMs) and ECs are the most abundant cell types [122,123,124]. Although CM has gained an important consideration in experimental MI studies, EC remains underappreciated. Recently, the role of EC has been underlined in post-infarction remodeling has emerged as a potential target for cardioprotection in ischemia/reperfusion injury [125,126]. A further step is to better understand how ECs contribute to MI. Such evidence is required if we are to fulfil our purpose of selectively targeting ECs in the future, which might open a new paradigm for MI treatment.

With this purpose, our recent data highlighted the importance of IDO expressed in EC in the modulation of cardiac remodeling and function following experimental MI [75]. In cardiac ischemic mouse model, IDO activity as assessed by Kyn/Trp ratio was markedly increased in the heart at day 1 after MI. This may depend on the induction of ischemia-associated inflammatory cytokines [127]. Advances in the understanding of the role of IDO in MI have led us to consider it deleterious. This observation was supported by the finding that loss of function of IDO as well as pharmacological inhibition of its activity by 1MT attenuated cardiac deleterious remodeling as well as cardiac dysfunction after MI. It is established that inflammation plays a key role in the progression of MI. Hence, its modulation could be responsible for infarct size reduction and cardiac dysfunction attenuation observed in IDO KO mice. Surprisingly, we did not observe any differences in the number of infiltrating cardiac inflammatory cells between *Ido-1^−/−^* and WT mice. Consistently, non-bone marrow (BM)-derived cells expressing IDO but not BM-derived cells were involved in the observed effects. The absence of a marked contribution of inflammatory cells in IDO-mediated effects could be due to the fact that they are not the major sources of the enzyme in the heart after MI. Moreover, the use of distinct specific IDO KO in CM, SMC, and EC allowed us to identify the importance of IDO expressed in EC to convey its harmful effects on cardiac remodeling and function. This is consistent with the observation that IDO expression and activity are more important in cardiac ECs than CMs or leukocytes. Mechanistically, we showed that Kyn mediates IDO deleterious effects given that Kyn supplementation to *Ido-1^−/−^* mice altered LV remodeling and led to cardiac dysfunction after MI. It also increased cell apoptosis in the infarcted heart as well as in vitro CM apoptosis. AHR may convey Kyn effect on CM apoptosis since its blockade abolished Kyn-induced pro-apoptotic effects, most likely through the reduction of ROS production. Therefore, our findings highlighted the importance of paracrine effects of Kyn released by EC on CM apoptosis.

Altogether, these findings exemplified a fundamental advance in our understanding of the cellular mechanisms consequent to MI which are likely to be governed by ECs. We pinpointed the communication between EC and CM and considered it as a fundamental player in cardiac remodeling and function, which supports the concept that healthy ECs are important for CM function, which in turn contributes to cardioprotection.

Likewise, in the context of experimental ischemia reperfusion (I/R) kidney injury, it has been shown an increased IDO expression, whereas IDO inhibition ameliorated kidney injury and preserved renal function [128]. Mechanistically, this has been associated to the activation of GCN2 kinase, which promotes cell apoptosis, and to the deleterious effects of AHR activation by Kyn [129].

Beyond KP contribution, evidence has proved that the 5-HT pathway is also implicated in MI. An early study showed that 5-HT enhanced neutrophil recruitment to sites of inflammation and tissue damage [130]. More recently, Mauler and coworkers provided convincing evidence showing an important role of platelet-derived 5-HT in aggravating myocardial I/R injury through neutrophil activation. This study suggests a preeminent role of 5-HT in neutrophil degranulation as well as in increasing the surface expression of leukocyte adhesion molecule CD11b and facilitating the release of myeloperoxidase (MPO), known to be involved in tissue damage. The inhibition of 5-HT reuptake, through the use of SSRIs, was beneficial as it reduced neutrophil degranulation and preserved cardiac function. These inhibitors act by increasing serotonergic transmission in brain which helps to treat depression. Their action also affects platelets by depleting them from serotonin. Since depression has been linked to increased risk of MI, SSRIs were believed to be protective against CVD where platelets are important contributors [131,132]. In line with these results, patients with depression treated with 5-HT reuptake inhibitors had lower neutrophil and CD11b surface expression levels as well as decreased MPO levels in blood, which is indicative of reduced inflammation [133]. Moreover, in a nested case-control study, the results showed a favorable role of strong 5-HT reuptake inhibitors in decreasing ischemic stroke incidence compared with the current use of weak inhibitors [134]. In a more recent study, the inhibition of Serotonin 2B receptor (5-HT_2B_) signaling preserved cardiac structure and function through inhibiting the excessive fibrotic process of scar formation after MI [135]. Taken together, these studies strongly suggest a deleterious role of 5-HT in MI. Therefore, deleting the monoamine through particularly the use of SSRIs may be beneficial in the early stage after MI.

Unlike 5-HT, melatonin has been shown to exert a cardioprotective role in acute MI. It acts against apoptosis and oxidative stress, promotes autophagic cell repair, enhances mitochondrial function and favorably modulates inflammatory response [113]. In recent years, more has been elucidated concerning the mechanisms that explain melatonin effects. Zhou et al. showed that melatonin suppressed platelet activity following cardiac I/R by restoring the peroxisome proliferator-activated receptor γ (PPARγ) content which in turn blocked FUN14 domain containing 1 (FUNDC1)-required mitophagy, which is important for platelet activation. Their results showed that genetic ablation of platelet-PPARγ reversed the beneficial effects of melatonin [136]. Another melatonin-mediated signaling pathway targeting mitochondria has been also highlighted in cardiac I/R. According to Zhang and colleagues, optic atrophy 1 (OPA1) mRNA and protein levels were significantly reduced in mice subjected to cardiac I/R compared to sham mice and this was reversed by melatonin. Moreover, melatonin treatment upregulated OPA1, which has as consequences sustained CM mitochondrial function and therefore improved cardiac function after cardiac I/R. In this study, melatonin was suggested to modulate OPA1 by up-regulating the AMP-activated protein kinase (AMPK) signaling pathway [137]. Human investigation reported that low circulating melatonin levels were associated with aggravated heart dysfunction in patients with post-acute MI [138]. Therefore, melatonin supplementation may represent a promising approach against various cardiovascular pathologies.

Altogether, these findings pinpoint Trp catabolic pathways as novel therapeutic targets in order to protect against harmful consequences of MI.

## 5. Therapeutic Approaches

Despite the significant reduction in cardiovascular events thanks to LDL-cholesterol lowering drugs, patients with CVD still exhibit residual cardiovascular risk [139]. The contributors of this residual risk, which underpins CVD development, are broad and their interactions are complex. Inflammation has been identified as a key actor involved at different steps of CVD development. This may cause what is called the residual inflammatory risk that may contribute to CVD regardless of lipid status. Recent studies showed that targeting inflammation may represent a promising therapeutic approach to reduce acute CV events [8,9]. Thus, a combination therapy, including lipid-lowering drugs together with targeting inflammation, could represent an attractive therapy. However, targeting inflammation may result in secondary side effects such as an increased infection risk. Therefore, acting on factors upstream of inflammation may represent a more attractive therapeutic avenue insofar as it may entail less perturbation of host defenses.

As such, diet modification may have favorable impacts on inflammation [140]. Given the close link between Trp metabolism and inflammation, targeting this pathway may reduce inflammation and therefore CVD.

Trp undergoes an extensive and complex metabolism along several pathways, resulting in many bioactive molecules acting in various organs through different mechanisms. Disruptions in Trp metabolism are reported in several diseases, including neurological, metabolic, psychiatric, intestinal disorders, and CVD, paving the way to develop drugs to target it.

IDO has emerged as a key extrahepatic enzyme that directs Trp catabolism toward KP. IDO has been implicated in a broad range of pathologies and IDO inhibitor has been used in clinical trials. However, the use of the IDO inhibitor has not been shown to be effective in a randomized phase III study in cancer [141]. One possibility is to re-purpose this inhibitor in other pathological contexts. In CVD, no randomized controlled trials of IDO inhibitors to prevent or treat CHD have been conducted. This is due to lack of mechanistic studies that may hinder clinical trials in this field.

Nevertheless, it should be kept in mind that IDO affects many biological pathways, thus inhibition of this enzyme may not be the appropriate strategy. In this context, studying the role of IDO downstream enzymes and testing their inhibitors may represent an interesting eventuality since it pinpoints a specific metabolic pathway. In this regard, inhibition of KMO enzyme which is directly involved in Quina production and indirectly involved in controlling Kna metabolite, has been considered as a therapeutic option, especially in neurological diseases [142]. Thus, studying the role of the Trp- catabolizing enzymes in different pathological settings including CVD, and eventually inhibiting the enzyme activity may be a new direction for therapy.

In addition to IDO and its downstream enzymes, other Trp-catabolizing pathways have been shown to play a significant role in CVD. Particularly, 5-HT has been shown to exert a pathogenic role in MI. Interestingly, a novel drug class which cannot pass the blood–brain barrier to selectively block peripheral 5-HT synthesis has been approved for the treatment of carcinoid syndrome [143]. Such inhibitors need to prove their efficacy for the treatment of other diseases including CVD in future preclinical and clinical studies. Besides, supplementation with melatonin, due to its well-known documented anti-inflammatory benefits, along with its very high safety profile even when used in high doses, seems to be an interesting therapeutic avenue [144]. Given the proven beneficial actions of melatonin in multiple organs, it would be interesting to test the effects of its supplementation in CVD.

However, before developing such potential treatments, it is essential to have a more complete and mechanistic understanding of the role of the different actors involved in Trp catabolism, in order to modulate them for treatment, e.g., of cardiometabolic diseases.

Besides, accumulating evidence supports the close interaction between intestine, microbiota, and immune system. Given the importance of Trp metabolism on intestinal homeostasis along with its close interaction with gut microbiota, this should be taken in account in future therapeutic considerations. Indeed, changes in the composition of the gut microbiota may predispose the gut into an inflammatory state leading to heightened systemic inflammation, and thereby CVD development.

Therefore, understanding the impact of Trp metabolism on the complex dialogue between the host and the gut microbiota now appears necessary for the development of new therapeutic approaches to positively modulate the microbiota and the treatment of diseases. Current data demonstrate the importance of indole metabolites in maintaining the integrity of the intestinal epithelial barrier in inflammatory bowel disease and metabolic syndrome. It is therefore necessary to identify the bacteria permitting this indole production and to clearly define the specific indoles allowing the activation of AHR. Although several bacteria such as *Lactobacilli reuteri* are capable of producing indoles [35], others may exist, and they need to be identified. Thus, the pathophysiological implications of AHR activation by tryptamine and the various metabolites of the indole pathway in the gastrointestinal tract remain to be established. Moreover, further studies are needed to determine their involvement in multifactorial diseases such as cardiovascular diseases. One of the main achievements would be to identify the best way to modulate the catabolism of Trp to improve dysbiosis and treat cardiometabolic diseases. In particular, additional studies are needed to determine whether indole derivatives produced by bacteria or the direct administration of these metabolites will have a beneficial effect on inflammatory diseases characterized by an alteration of the intestinal barrier. Likewise, future studies are needed to determine the therapeutic efficacy of the combined or separate use of these bacteria and/or metabolites of the indole pathway. This may represent the first step towards personalized medicine in the field of CVD.

## 6. Conclusions

KP, a major Trp breakdown pathway, which is induced by inflammatory factors such as IFN-γ, has been shown to be increased in various pathological conditions including CVD. Importantly, an elevated Kyn/Trp ratio, which is indicative of KP activation, has been shown to be predictive of a higher likelihood of cardiovascular morbidity and mortality. Future experimental studies would be instrumental to determine whether the KP increase should be considered only as biomarker or whether the different actors involved in KP could modulate inflammation and thereby CVD. More research is needed to gain comprehensive insight into the function of the KP along with the other Trp-degrading pathways in CVD. Specifically, future studies should focus on the interplay between Trp catabolism and microbiota and how this mutualistic interaction could impact on CVD. This comprehensive overview can provide a basis for more successful, precise, biologically grounded therapeutic protocols to combat CVD. As such, interventions will most likely require a profound knowledge of disease development combined with a personalized monitoring of Trp metabolism role so as not to risk a complete blocking of activities which are potentially necessary for health homeostasis.

## Data Availability

Not applicable.
